# Artificial intelligence–enabled histology exhibits comparable accuracy to pathologists in assessing histological remission in ulcerative colitis: a systematic review, meta-analysis, and meta-regression

**DOI:** 10.1093/ecco-jcc/jjae198

**Published:** 2025-01-01

**Authors:** Miguel Puga-Tejada, Snehali Majumder, Yasuharu Maeda, Irene Zammarchi, Ilaria Ditonno, Giovanni Santacroce, Ivan Capobianco, Carlos Robles-Medranda, Subrata Ghosh, Marietta Iacucci

**Affiliations:** APC Microbiome Ireland, College of Medicine and Health, University College Cork (UCC), Cork, Ireland; Instituto Ecuatoriano de Enfermedades Digestivas (IECED), Guayaquil, Ecuador; APC Microbiome Ireland, College of Medicine and Health, University College Cork (UCC), Cork, Ireland; APC Microbiome Ireland, College of Medicine and Health, University College Cork (UCC), Cork, Ireland; APC Microbiome Ireland, College of Medicine and Health, University College Cork (UCC), Cork, Ireland; APC Microbiome Ireland, College of Medicine and Health, University College Cork (UCC), Cork, Ireland; APC Microbiome Ireland, College of Medicine and Health, University College Cork (UCC), Cork, Ireland; APC Microbiome Ireland, College of Medicine and Health, University College Cork (UCC), Cork, Ireland; Instituto Ecuatoriano de Enfermedades Digestivas (IECED), Guayaquil, Ecuador; APC Microbiome Ireland, College of Medicine and Health, University College Cork (UCC), Cork, Ireland; APC Microbiome Ireland, College of Medicine and Health, University College Cork (UCC), Cork, Ireland

**Keywords:** artificial intelligence, histological assessment, ulcerative colitis

## Abstract

**Background and Aims:**

Achieving histological remission is a desirable emerging treatment target in ulcerative colitis (UC), yet its assessment is challenging due to high inter- and intraobserver variability, reliance on experts, and lack of standardization. Artificial intelligence (AI) holds promise in addressing these issues. This systematic review, meta-analysis, and meta-regression evaluated the AI’s performance in assessing histological remission and compared it with that of pathologists.

**Methods:**

We searched Medline/PubMed and Scopus databases from inception to September 2024. We included studies on AI models assessing histological activity in UC, with or without comparison to pathologists. Pooled performance metrics were calculated: sensitivity, specificity, positive and negative predictive value (PPV and NPV), observed agreement, and F1 score. A pairwise meta-analysis compared AI and pathologists, while sub-meta-analysis and meta-regression evaluated heterogeneity and factors influencing AI performance.

**Results:**

Twelve studies met the inclusion criteria. AI models exhibited strong performance with a pooled sensitivity of 0.84 (95% CI, 0.80–0.88), specificity 0.87 (0.84–0.91), PPV 0.90 (0.87–0.92), NPV 0.80 (0.71–0.88), observed agreement 0.85 (0.82–0.89), and F1 score 0.85 (0.82–0.89). AI models demonstrated no significant differences with pathologists for specificity, observed agreement, and F1 score, while they were outperformed by pathologists for sensitivity and NPV. AI models for the adult population were linked to reduced heterogeneity and enhanced AI performance at meta-regression.

**Conclusions:**

AI shows significant potential for assessing histological remission in UC and performs comparably to pathologists. Future research should focus on standardized, large-scale studies to minimize heterogeneity and support widespread AI implementation in clinical practice.

## Graphical Abstract

**Graphical Abstract:**
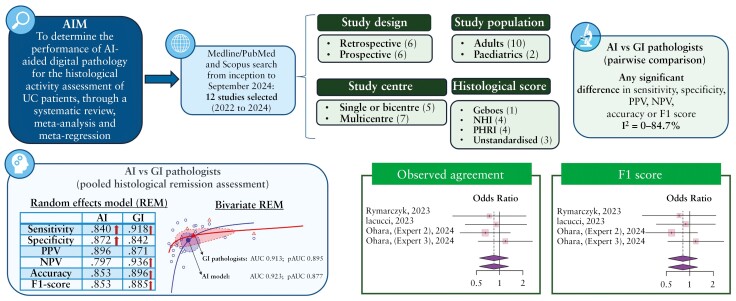

## 1. Introduction

Endoscopic remission is the key long-term treatment target in inflammatory bowel disease (IBD) and it is associated with a reduced risk of major adverse outcomes.^1^ Nonetheless, histological activity can persist even when the mucosa appears endoscopically normal. Approximately 30% of ulcerative colitis (UC) patients with clinical and endoscopic remission still exhibit histological activity.^2^ UC patients in endoscopic remission with ongoing histological activity have a higher risk of relapse.^3,4^ Conversely, no clear association between histological activity and relapse risk has been observed in Crohn’s disease (CD) patients with endoscopic remission.^5^ Thus, histological remission has emerged as a more robust therapeutic target in UC, as it significantly predicts disease course and favorable outcomes.^6^

However, assessing histological remission presents several challenges, including the absence of uniformly acknowledged definitions, the lack of standardized histopathology reports, the high intra- and interobserver variability even among experienced pathologists, and the presence of multiple histological scores, some not validated. A recent global survey found that most histological reports are descriptive and nonstandardized despite the critical role of histological activity in patient management.^7^ A European Crohn’s and Colitis Organization (ECCO) expert panel has worked to harmonize and standardize UC histopathology, establishing common definitions and encouraging validated scoring systems, especially in clinical trials.^8,9^

Given these challenging scenarios, introducing artificial intelligence (AI) by incorporating digital slides into whole-slide images (WSI) may hold great promise.^10,11^

The AI can potentially evaluate the histological activity objectively and accurately in IBD, overcoming the inter- and intraobserver variability, the need for uniform expertise, and time constraints. Indeed, AI models have demonstrated remarkable performance in assessing histological activity in UC.^12–23^ Such an automated approach could be particularly beneficial in clinical trials by providing standardized central readings and objectively evaluating responses to therapy while reducing time and costs by resolving discrepancies.^24^ Moreover, AI has shown promising capabilities in predicting 12-month relapse risk.^13,16^ It is particularly compelling for clinical practice as it can guide disease management and represents a significant step toward personalized medicine.^25^

The following systematic review, meta-analysis, and meta-regression aim to (1) estimate the diagnostic performance of AI-enabled models in the histological activity assessment of UC patients and (2) compare the diagnostic performance of AI-enabled models versus humans in terms of gastrointestinal (GI) pathologists or non-GI pathologists, when available.

## 2. Methods

### 2.1. Protocol registration

This meta-analysis adhered to the Preferred Reporting Items for Systematic Reviews and Meta-Analyses extension statement for systematic reviews (PRISMA) guidelines,^26^ the Meta-Analysis of Observational Studies in Epidemiology (MOOSE) recommendations,^27^ and current standards for reporting diagnostic test accuracy of meta-analyses.^28^ The protocol was registered with PROSPERO (ID CRD42024555452). Six authors (M.P-T., S.M., Y.M., I.Z., I.D., and G.S.) independently conducted all literature search, study selection, quality assessment, and data extraction according to a standardized protocol. Discrepancies were resolved through discussion and consensus among the 6 authors.

### 2.2. Search strategy

We systematically searched the Medline/Pubmed and Scopus databases from inception to September 2024, with English as the only language restriction and no limits on ethnicity or region. Our search strategy included a combination of Medical Subject Headings (MeSH) and keywords related to AI, Histology, and IBD, using Boolean operators: “artificial intelligence,” “deep learning,” “machine learning,” “convolutional neural network,” “computer-aided assessment,” “algorithm,” “histology,” “histological,” “histopathology,” “histopathological,” “biopsy,” “biopsies,” “slide,” “slides,” “pathology,” “pathologic,” “ulcerative colitis,” “UC,” We also utilized references from previous reviews and meta-analyses to ensure comprehensive coverage.

### 2.3. Study selection

After excluding duplicates, 3 independent, experienced reviewers (S.M., Y.M., and I.Z.) screened titles and abstracts for inclusion and exclusion criteria. Full-text reviews were conducted for abstracts meeting inclusion criteria or unclear cases. A fourth independent and experienced reviewer (M.P.-T.) resolved disagreements. Citations were managed using the EPPI-Reviewer software v4.0 (University College London, UK; ID 49306).

### 2.4. Inclusion and exclusion criteria

We included full papers that fulfilled the following criteria:

- **Population:** Studies involving UC patients, regardless of study design (retrospective or prospective), number of participating centers (single, bicentric, or multicentric), and age group of the studied population (adults or pediatrics).
- **Intervention:** Any WSI-based AI model developed for UC histological activity assessment, regardless of its development stage (training, testing, or validation).
- **Comparison:** Studies with or without comparing AI models and pathologists’ diagnostic performance.
- **Outcome data:** Reports on AI diagnostic performance and, if available, pathologist performance.

The exclusion criteria were as follows: (1) duplicates, (2) non-English studies, (3) nonhuman studies, (4) consensus papers, guidelines, letters to the Editor, book chapters or case reports, (5) literature or systematic reviews, (6) non-AI-enabled IBD models, (7) studies lacking extractable outcome data.

### 2.5. Quality of evidence

Two independent reviewers (M.P.-T. and S.M.) assessed study quality using the Quality Assessment of Diagnostic Accuracy Studies (QUADAS)-2. The quality evaluation criteria included patient selection, index test, reference standard, flow, and timing. Discrepancies were resolved by discussion and consensus.

### 2.6. Data extraction

Four independent reviewers (Y.M., I.Z., I.D., and G.S.) extracted data from qualifying studies, including (1) author’s information; (2b) publication date; (3) country; (4) sample size; (5) observer type (AI model, GI or non-GI pathologists, in agreement with the definition of each study); (6) study design; (7) number of participating centers (single, bicentric, or multicentric); (8) population age group (adults or pediatrics, defined as up to 18 years old); (9) AI model stage (training, testing or validation); (10) parameters of histological activity; and (11) data from contingency tables. Data were extracted per each retrieved contingency table.

### 2.7. Outcomes measurement

This meta-analysis pooled AI diagnostic performance to assess UC histological activity. When available, GI or non-GI pathologists’ diagnostic performance was evaluated. Metrics included sensitivity (or recall), specificity, positive predictive value (PPV, or precision), negative predictive value (NPV), observed agreement (or accuracy), and F1 score.

### 2.8. Data synthesis

#### 2.8.1. Meta-analyses and meta-regressions

We conducted meta-analyses and meta-regressions using common, random, and bivariate random effects models to determine and differentiate the pooled diagnostic performance per observer regarding sensitivity, specificity, PPV, NPV, observed agreement, F1 score, and summary receiver-operating characteristic curve (SROC) derived from contingency tables. Publication bias was detected using corrected funnel plots and Egger’s test.^29^ The *I*^2^ statistic was used to test for heterogeneity in the studies within the common effects model analyses. Heterogeneity was low when *I*^2^ was between 0% and 25%, moderate up to 50%, considerable up to 75%, and high above 75%.^30^ For random effect models, heterogeneity was assessed through τ and τ^2^. A τ < 0.2 and τ^2^ < 0.04 defined low heterogeneity, τ = 0.2–0.5 and τ^2^ = 0.04–0.25 moderate heterogeneity, and τ > 0.5 and τ^2^ > 0.25 high heterogeneity. For the bivariate random effect model analysis, heterogeneity was determined with the *I*^2^ Holling-adjusted approaches.

#### 2.8.2. Pairwise meta-analyses

A pairwise comparison of diagnostic performance between AI models versus GI or non-GI pathologists, when available, was performed using common and random effects models. Diagnostic ODDS RATIO (OR) and 95% confidence interval (CI) were calculated, with results depicted in forest plots.

#### 2.8.3. Additional analyses

Additional sub-meta-analyses and meta-regressions were conducted exclusively among AI models to explore heterogeneity sources and factors affecting the performance metrics. The variables of interest included (1) study design (retrospective or prospective), (2) center type (single or bicentric vs multicentric), (3) age group (adults or pediatrics), and (4) AI model stage (training, testing, or validation).

#### 2.8.4. Technical considerations

Data were analyzed by a gastroenterologist and certified biostatistician (M.P.-T.) using R v4.0 (R Foundation for Statistical Computing). A *P*-value <.05 was considered as statistically significant.

## 3. Results

### 3.1. Study selection and characteristics

**Figure 1.**
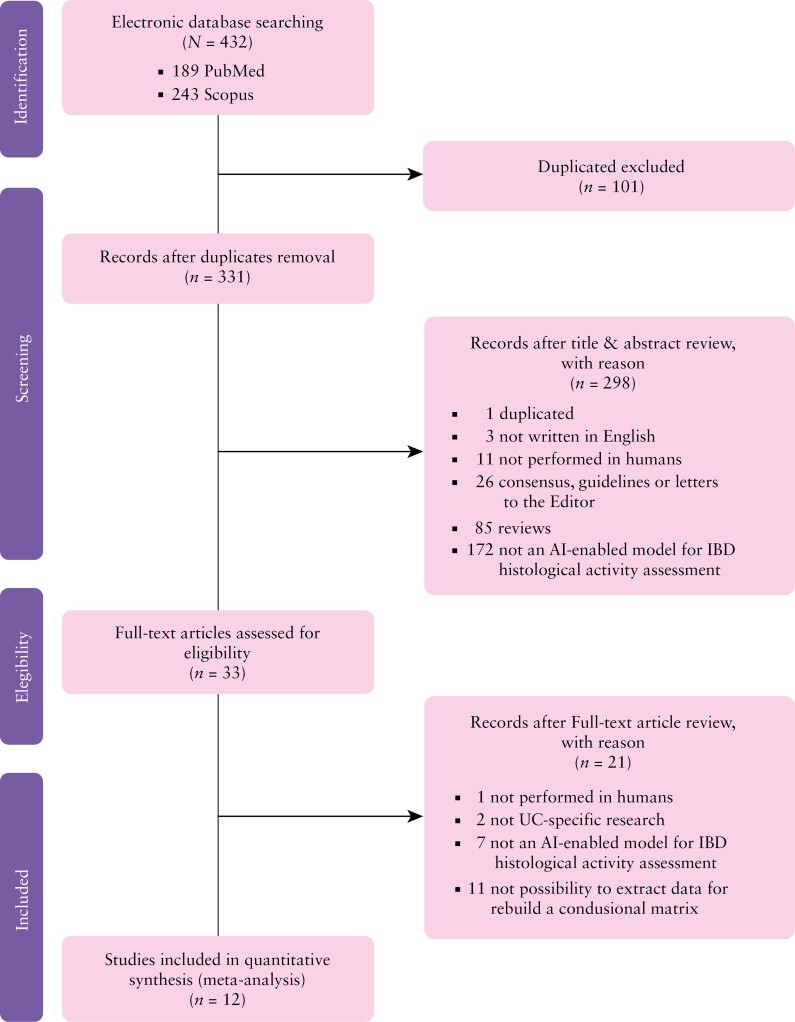
Literature review flowchart.

A total of 432 records were identified through electronic database search. After removing duplicates (101), 331 titles and abstracts were screened. Of these, 298 were excluded based on predefined criteria. During the full-text review of the remaining 33 articles, 21 were excluded, leaving 12 studies for inclusion in the meta-analysis (Figure 1).^12–23^

The studies included originated from United States (4), United Kingdom (3), Japan (2), France (1), Poland (1), and Spain (1). 6/12 studies were prospective. Most studies were multicentric (7/12), conducted in adults (10/12), and confirmed the AI model’s diagnostic performance in a testing cohort (9/12). The histological activity was assessed using validated scores including Geboes score, Nancy Histologic index, and PICaSSO Histological Remission Index in 9/12 studies, while 3/12 studies did not use any validated index. Study characteristics are detailed in Table 1 and summarized in Supplementary Table 1. Data from the 12 selected studies allowed for reconstructing 30 contingency tables, with 25 based on AI models and 5 on pathologists. Data from GI pathologists came from 3 studies that defined them as experts but without a standard definition of expertise.^15,18,20^ No data were available on non-GI pathologists. The analysis included 4720 observations from AI models and 547 from GI pathologists. No observation was found for non-GIpathologists.

**Table 1.** Characteristics of selected studies for the systematic review and meta-analysis.

Author (year)	Country	Sample size	Study design	No. of centers	Population age group	Type of disease	Model stage	Observer	Histological parameter	Sensitivity (recall)	Specificity	PPV (precision)	NPV	Observed agreement (accuracy)	F1 score
Del Amor et al. (2022)^12^	Spain	100	Prospective	Multicentric	Adults	UC	Testing	AI model	PHRI	0.96	0.83	0.90	0.96	0.91	0.93
Gui et al. (2022)^1^^3^	UK	215	Prospective	Multicentric	Adults	UC	Testing	AI model	PHRI	0.78	0.91	0.84	0.86	0.86	0.81
	UK	214	Prospective	Multicentric	Adults	UC	Testing	AI model	PHRI	0.82	0.94	0.89	0.89	0.89	0.86
	UK	93	Prospective	Multicentric	Adults	UC	Validation	AI model	PHRI	0.67	0.89	0.80	0.81	0.81	0.73
	UK	93	Prospective	Multicentric	Adults	UC	Validation	AI model	PHRI	0.81	0.91	0.85	0.88	0.87	0.83
Ohara et al. (2022 )^14^	Japan	114	Retrospective	Single or bicentric	Adults	UC	Validation	AI model	Goblet cells	0.83	0.99	0.91	0.98	0.97	0.87
	Japan	114	Retrospective	Single or bicentric	Adults	UC	Validation	AI model	Goblet cells	0.88	0.63	0.93	0.45	0.84	0.91
	Japan	114	Retrospective	Single or bicentric	Adults	UC	Validation	AI model	Goblet cells	0.93	0.96	0.96	0.93	0.95	0.95
Vande Casteele et al. (2022)^1^^5^	USA	88	Retrospective	Single or bicentric	Adults	UC	Testing	AI model	Eosinophils	0.86	0.91	0.90	0.87	0.89	0.88
Iacucci et al. (2023)^1^^6^	UK	42	Prospective	Multicentric	Adults	UC	Training	AI model	PHRI	0.75	0.96	0.92	0.86	0.88	0.83
	UK	375	Prospective	Multicentric	Adults	UC	Testing	AI model	PHRI	0.89	0.85	0.77	0.93	0.86	0.83
	UK	154	Prospective	Multicentric	Adults	UC	Validation	AI model	PHRI	0.92	0.82	0.95	0.73	0.90	0.93
	UK	47	Prospective	Multicentric	Adults	UC	Validation	AI model	PHRI	0.87	0.44	0.75	0.64	0.72	0.81
	UK	47	Prospective	Multicentric	Adults	UC	Validation	GI Pathologist	PHRI	0.90	0.44	0.76	0.70	0.74	0.82
Najdawi et al. (2023)^1^^7^	USA	627	Prospective	Multicentric	Adults	UC	Validation	AI model	NHI	0.98	0.93	0.97	0.96	0.96	0.97
Rymarczyk et al. (2023)^1^^8^	Poland	817	Retrospective	Multicentric	Adults	UC	Training	AI model	GS	0.96	0.81	0.91	0.91	0.91	0.94
	Poland	95	Retrospective	Multicentric	Adults	UC	Testing	AI model	GS	0.97	0.73	0.92	0.89	0.92	0.95
	Poland	95	Retrospective	Multicentric	Adults	UC	Testing	GI Pathologist	GS	1.00	0.73	0.92	1.00	0.94	0.96
Rubin et al. (2024)^1^^9^	USA	148	Prospective	Multicentric	Adults	UC	Testing	AI model	NHI	1.00	0.88	0.95	1.00	0.96	0.98
Ohara et al. (2024)^20^	Japan	135	Prospective	Single or bicentric	Adults	UC	Testing	AI model	PHRI	0.71	0.97	0.95	0.82	0.86	0.81
	Japan	135	Prospective	Single or bicentric	Adults	UC	Testing	AI model	PHRI	0.81	0.98	0.96	0.89	0.91	0.88
	Japan	135	Prospective	Single or bicentric	Adults	UC	Testing	GI pathologist	PHRI	0.83	0.96	0.94	0.88	0.90	0.88
	Japan	135	Prospective	Single or bicentric	Adults	UC	Testing	GI pathologist	PHRI	0.88	0.91	0.84	0.93	0.90	0.86
	Japan	135	Prospective	Single or bicentric	Adults	UC	Testing	GI pathologist	PHRI	0.94	0.87	0.81	0.96	0.90	0.87
Peyrin-Biroulet et al. (2024)^21^	France	40	Retrospective	Single or bicentric	Adults	UC	Testing	AI model	NHI	0.79	0.96	0.92	0.89	0.90	0.85
Reigle et al. (2024)^2^^2^	USA	150	Retrospective	Single or bicentric	Pediatrics	UC	Validation	AI model	Eosinophils	0.96	0.77	0.77	0.96	0.85	0.85
Liu et al. (2024)^23^	UK	292	Retrospective	Multicentric	Pediatrics	UC	Training	AI model	NHI	0.84	0.94	0.91	0.42	0.90	0.87
	UK	292	Retrospective	Multicentric	Pediatrics	UC	Training	AI model	NHI	0.47	0.50	0.59	0.10	0.48	0.48
	UK	113	Retrospective	Multicentric	Pediatrics	UC	Testing	AI model	NHI	0.78	0.91	0.84	0.79	0.85	0.82
	UK	113	Retrospective	Multicentric	Pediatrics	UC	Testing	AI model	NHI	0.42	0.43	0.53	0.36	0.48	0.43

Abbreviations: AI, Artificial Intelligence; CD, Crohn’s disease; GI, gastrointestinal; GS, Geboes Score; PPV, positive predictive value; UC, ulcerative colitis; UK, United Kingdom.

**Figure 2.**
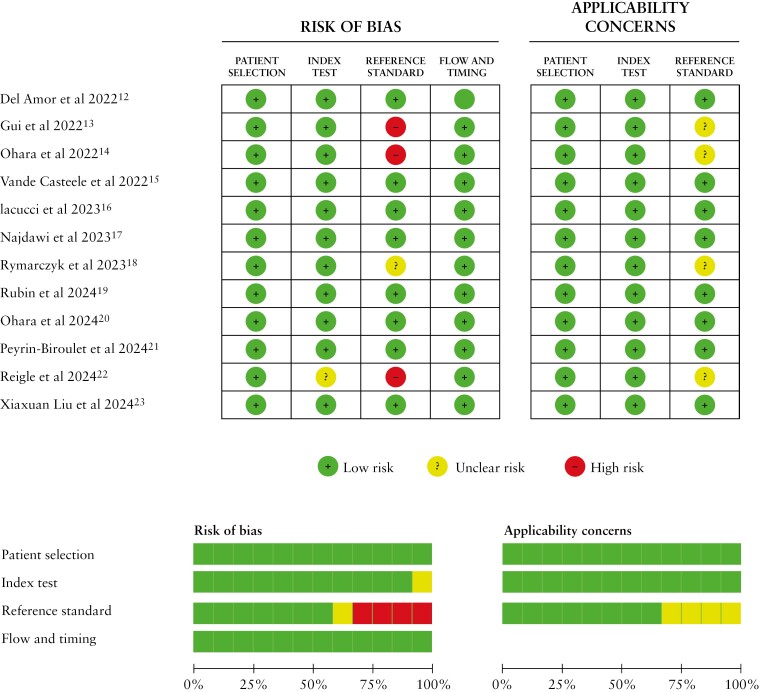
Assessment of risk of bias of studies. QUADAS-2 tool. QUADAS-2, Quality Assessment of Diagnostic Accuracy Studies-2.

According to the quality analysis through the QUADAS-2 tool, selected papers presented a low risk of bias in terms of patient selection (100%), index test (91.7%), and flow and timing (100%). Cause of the diversity of validated and nonvalidated indices between selected papers, a 25% high risk of bias was determined. There was a 100% low risk of applicability concerns for patient selection or index test but a 33.3% unclear risk of bias for reference standard (Figure 2).

### 3.2. AI-aided histology performance in assessing disease activity or remission

In the common effect model, the AI models demonstrated notable performance metrics in assessing histological activity despite a high level of heterogeneity (Table 2).

**Table 2.** Sub-meta-analysis by different observers: artificial intelligence models versus gastrointestinal (GI) pathologists.

Subgroup	No. of studies	Common effect model					Random effects model			
		Proportion (95% CI)	*Q*	*I* ^2^ (%)	*Q* between groups (*P*-value)	*Q* within groups (*P*-value)	Proportion (95% CI)	τ^2^	τ	Q between groups (*P*-value)
Sensitivity (recall)										
AI model	25	0.9565 (0.9495–0.9636)	517.17	95.4	8.31	539.16	0.8401 (0.8001–0.8801)	0.0082	0.0904	3.57
Pathologists	5	0.9838 (0.9667–1.0009)	21.99	81.8	(.0039)	(<.0001)	0.918 (0.8478–0.9882)	0.0048	0.0696	(.0589)
Specificity										
AI model	25	0.9362 (0.9258–0.9465)	206.29	88.4	1.57	230.93	0.8722 (0.8373–0.907)	0.0058	0.0763	.34
Pathologists	5	0.9153 (0.8843–0.9462)	24.65	83.8	(.2102)	(<.0001)	0.8422 (0.7481–0.9364)	0.0081	0.0901	(.5592)
PPV (precision)										
AI model	25	0.9532 (0.9454–0.961)	177.4	86.5	10.24	188.02	0.8955 (0.8686–0.9224)	0.0031	0.0557	.48
Pathologists	5	0.8934 (0.8576–0.9292)	10.62	62.3	(.0014)	(<.0001)	0.8713 (0.8085–0.934)	0.003	0.055	(.4868)
NPV										
AI model	25	0.8835 (0.8727–0.8943)	1323.52	98.2	13.87	1332.27	0.797 (0.7126–0.8815)	0.0437	0.2091	7.86
Pathologists	5	0.9412 (0.9128–0.9696)	8.75	54.3	(.0002)	(<.0001)	0.9356 (0.8883–0.9829)	0.0014	0.0377	(.005)
Observed agreement (accuracy)										
AI model	25	0.9111 (0.9033–0.9188)	474.01	94.9	.43	482.19	0.8531 (0.8159–0.8904)	0.0081	0.0898	2.64
Pathologists	5	0.9023 (0.8776–0.9271)	8.18	51.1	(.5098)	(<.0001)	0.8966 (0.8597–0.9335)	9.00E-04	0.0294	(.1043)
F1 score										
AI model	25	0.9317 (0.9252–0.9381)	466.69	94.9	1.44	484.66	0.8531 (0.82–0.8861)	0.0059	0.0769	.94
Pathologists	5	0.9171 (0.8941–0.94)	17.97	77.7	(.23)	(<.0001)	0.8851 (0.8294–0.9407)	0.003	0.0547	(.3325)

Abbreviations: I2 (%): Heterogeneity Statistic-the proportion of the variance in observed effect is due to variance in true effects rather than sampling error; τ2; Tau Squared-Heterogeneity Score- between study variance in the meta-analysis.

**Figure 3.**
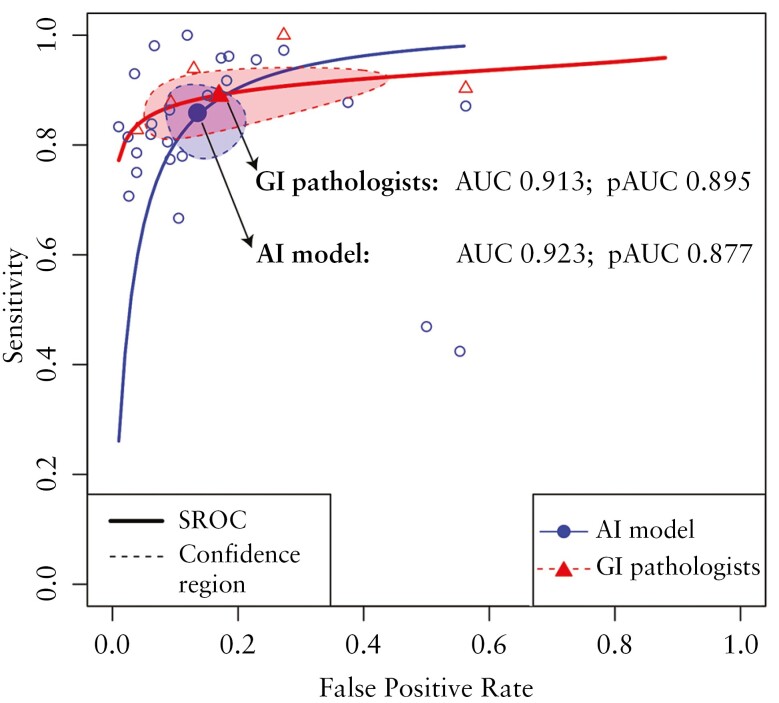
Summary receiver-operating characteristic curve (SROC) and partial area under the curve (pAUC) analyses, comparing the performance between artificial intelligence (AI) models versus gastrointestinal (GI) pathologists. The pAUC have been represented by circled areas, highlighted with dotted lines (confidence region).

Specifically, the metrics were as follows: sensitivity of 0.9565 (*I*^2^ = 95.4%), specificity of 0.9362 (*I*^2^ = 88.4%), PPV of 0.9532 (*I*^2^ = 86.5%), NPV of 0.8835 (*I*^2^ = 98.2%), observed agreement of 0.9111 (*I*^2^ = 94.9%), and F1 score of 0.9317 (*I*^2^ = 94.9%). When adjusted to the random effects model, the AI models maintained remarkable performance metrics with low heterogeneity, with a sensitivity of 0.8401 (τ^2^ 0.0082), specificity of 0.8722 (τ^2^ 0.0058), PPV of 0.8955 (τ^2^ 0.0031), NPV of 0.797 (τ^2^ 0.0437), observed agreement of 0.8531 (τ^2^ 0.0081), and F1 score of 0.8531 (τ^2^ 0.0059). Finally, the bivariate random effects model determined with a low heterogeneity (*I*^2^ = 7%–15.5%), a sensitivity, specificity, PPV, NPV, observed agreement, and F1 score of 0.854, 0.864, 0.729, 0.930, 0.861, and 0.788, respectively. The SROC was 0.923, with a partial area under the curve of 0.877 (Figure 3).

### 3.3. AI-aided histology models performance vs pathologists

When comparing the pooled outcomes of AI models (25 contingency tables) with those of pathologists (5 contingency tables) (Table 2), the common effect model showed that pathologists achieved the highest sensitivity (0.98 vs 0.96; *P* = .0039) and NPV (0.94 vs 0.88; *P* = .0.002). Conversely, no significant differences were found between AI models and pathologists for specificity (0.94 vs 0.92; *P* = .21), observed agreement (0.91 vs 0.90; *P* = .51), and F1 score (0.93 vs 0.92; *P* = 0.23). After adjusting for the random effects model, pathologists maintained a higher sensitivity (0.92 vs 0.84; *P* = .06) and NPV (0.94 vs 0.80; *P* = .005), while a nonsignificant difference was found for specificity, PPV, observed agreement, and F1 score. The meta-regression analysis showed that no significant difference is present for all performance measures between pathologists and AI models (Table 3). The bivariate random effects model determined a pooled pathologists’ performance of 0.891 for sensitivity, 0.831 for specificity, 0.693 for PPV, 0.946 for NPV, 0.849 for observed agreement, 0.779 for F1 score, 0.913 SROC, and 0.895 partial area under the curve (Figure 3), with a very low heterogeneity (*I*^2^ = 0.6%–1.7%).

**Table 3.** Meta-regression of the effect of heterogeneity in Artificial Intelligence (AI) diagnostic performance.

Covariate	Reference	Diagnostic performance parameter	Diagnostic performance difference (95% CI; *P*-value)	τ^2^	τ	Residual *I*^2^ (%)	*H* ^2^
Between AI models (*n* = 25) versus gastrointestinal pathologists (*n* = 5)							
Observer	Pathologist	Sensitivity	0.1055 (−0.0283, 0.2393; .1224)	0.008	0.0893	94.81	19.26
		Specificity	−0.029 (−0.1575, 0.0995; .6586)	0.006	0.0774	87.88	8.25
		PPV	−0.0346 (−0.1317, 0.0626; .4855)	0.0031	0.0557	85.11	6.71
		NPV	0.1558 (−0.1263, 0.4379; .279)	0.0394	0.1986	97.9	47.58
		Observed agreement	0.0427 (−0.0835, 0.1688; .5074)	0.0075	0.0866	94.19	17.22
		F1 score	0.0412 (−0.0711, 0.1534; .472)	0.0057	0.0758	94.22	17.31
Between AI models only (*n* = 25)							
Study design	Prospective	Sensitivity	0.0588 (−0.0602, 0.1778; .3326)	0.0092	0.0959	95.01	20.02
		Specificity	0.0876 (−0.018, 0.1932; .1041)	0.0068	0.0827	88.78	8.91
		PPV	0.0022 (−0.081, 0.0854; .9581)	0.0039	0.0628	86.74	7.54
		NPV	0.2164 (−0.0268, 0.4596; .0812)	0.0453	0.2129	98.13	53.5
		Observed agreement	0.0805 (−0.0284, 0.1894; .1474)	0.0087	0.0931	94.8	19.22
		F1 score	0.0359 (−0.0626, 0.1345; .4748)	0.0067	0.0817	94.52	18.25
**Number of centers**	Multicentric	Sensitivity	−0.0349 (−0.1594, 0.0896; .5828)	0.0081	0.0903	95.39	21.7
		Specificity	−**0.1166 (-0.2078, **−**0.0255; .0121)**	**0.0042**	**0.0651**	**83.48**	**6.05**
		PPV	−0.0502 (−0.1342, 0.0337; .2409)	0.0032	0.0568	86.82	7.59
		NPV	−0.1267 (−0.3917, 0.1382; .3485)	0.0471	0.217	98.19	55.29
		Observed agreement	−0.094 (−0.2104, 0.0225; .1136)	0.0087	0.0931	95.13	20.52
		F1 score	−0.0504 (−0.1528, 0.0519; .3341)	0.006	0.0775	94.93	19.72
Population age group	Pediatrics	Sensitivity	−**0.2401 (**−**0.3561, **−**0.1241; <.001)**	**0.0053**	**0.0731**	**93.08**	**14.45**
		Specificity	−**0.2076 (**−**0.3295, **−**0.0857; .0008)**	**0.0048**	**0.0695**	**86.53**	**7.42**
		PPV	−0.0824 (−0.1843, 0.0194; .1127)	0.0038	0.062	84.97	6.65
		NPV	−**0.4879 (**−**0.7146, **−**0.2611; <.001)**	**0.0245**	**0.1565**	**96.77**	**30.98**
		Observed agreement	−**0.2756 (**−**0.3822, **−**0.169; <.001)**	**0.0049**	**0.07**	**91.93**	**12.4**
		F1 score	−**0.1932 (**−**0.2962, **−**0.0903; .0002)**	**0.0045**	**0.0674**	**93.19**	**14.69**
AI model stage	Testing	Sensitivity	0.1249 (−0.138, 0.3877; 0.3518)	0.0159	0.1261	95.45	21.98
		Specificity	−0.0141 (−0.1896, 0.1615; 0.8753)	0.0061	0.0778	87.42	7.95
		PPV	0.0483 (−0.0957, 0.1922; 0.5112)	0.0037	0.0607	84.61	6.5
		NPV	−0.0298 (−0.4136, 0.354; 0.8791)	0.0363	0.1906	97.56	40.95
		Observed agreement	0.0613 (−0.1326, 0.2553; 0.5353)	0.0091	0.0952	94.48	18.1
		F1 score	0.0856 (−0.1044, 0.2755; 0.3771)	0.0084	0.0915	94.7	18.88
	Validation	Sensitivity	−0.0101 (−0.2314, 0.2112; 0.9288)	—	—	—	—
		Specificity	−0.0455 (−0.1924, 0.1014; 0.5441)	—	—	—	—
		PPV	0.0762 (−0.0412, 0.1936; 0.2033)	—	—	—	—
		NPV	−0.2901 (−0.6111, 0.0309; 0.0765)	—	—	—	—
		Observed agreement	−0.0488 (−0.2122, 0.1146; 0.5584)	—	—	—	—
		F1 score	0.022 (−0.1376, 0.1816; 0.7873)	—	—	—	—

Abbreviations: Diagnostic performance difference; performance measures between pathologists and AI models; H2: Statistic for assessing heterogeneity, a value of H2=1 indicates perfect homogeneity among studies; I2 (%): Heterogeneity Statistic; τ2; Tau Squared-Heterogeneity Score-between study variance in the meta-analysis.

#### 3.3.1. Pairwise comparison

Three of the 12 studies included at least 1 pairwise comparison between the diagnostic performance of an AI model and a pathologist. Of these studies, 1/3 were retrospective, 2/3 were multicentric, and all studies involved adults. All pairwise comparisons were conducted within testing or validation cohorts. Seven contingency tables were extracted, corresponding to outcomes from 7 AI models and 7 pathologists.

**Figure 4.**
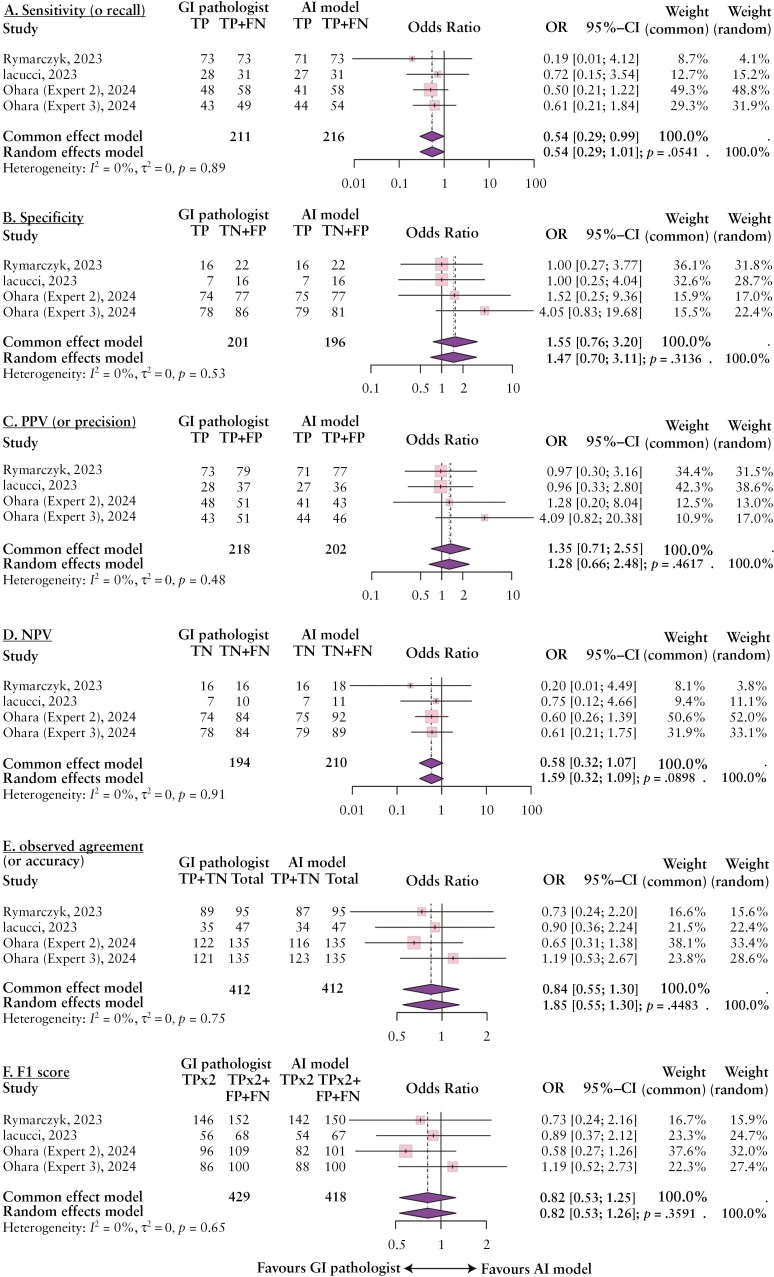
Forest plots illustrate diagnostic performance between Artificial Intelligence (AI) models vs gastrointestinal (GI) pathologists in terms of (A) sensitivity (or recall), (B) sensitivity, (C) positive predictive value (PPV or precision), (D) negative predictive value (NPV), (E) observed agreement (or accuracy), and (F) F1 score.

Figure 4 presents the forest plots of the pairwise meta-analysis contrasting the diagnostic performance of AI models with pathologists. Using the AI model as the diagnostic intervention and the pathologist as the diagnostic standard, the random effects model indicated comparable diagnostic performance between AI models and pathologists in terms of sensitivity (OR 0.54 [0.29–1.01]; *P* = .05) (Figure 4A), specificity (OR 1.47 [0.70–3.11]; *P* = .31) (Figure 4B), PPV (OR 1.28 [0.66–2.48]; *P* = .46) (Figure 4C), NPV (OR 0.59 [0.32–1.09]; *P* = .09) (Figure 4D), observed agreement (OR 0.85 [0.55–1.3]; *P* = .45) (Figure 4E), and F1 score (OR 0.82 [0.53–1.26]; *P* = .36) (Figure 4F). These results were obtained in low to considerable heterogeneity (*I*^2^ = 0%, 95% CI, 0%–84.7%).

### 3.4. AI-aided histology models performance and study heterogeneity—sub-meta-analyses and meta-regression

#### 3.4.1. Sub-metanalyses

A total of 25 contingency tables from the 12 studies included were subjected to a sub-meta-analysis based on study design (Supplementary Table 2), number of participating centers (Supplementary Table 3), population age group (Supplementary Table 4), and model stage (Supplementary Table 5).

The common effect model indicated higher heterogeneity when analyzing retrospective (*I*^2^ = 90.3%–99.1%) versus prospective studies (*I*^2^ = 76.5%–91.8%), multicentric (*I*^2^ = 85%–98.7%) versus single or bicentric (*I*^2^ = 48.7%–84.4%), pediatrics (*I*^2^ = 92%–99.4%) versus adults (*I*^2^ = 71.6 to 88.1%). Heterogeneity also decreased from training (*I*^2^ = 90.2%–99.6%) to testing (*I*^2^ = 82.9%–94.2%), and then to validation (*I*^2^ = 76.5%–88.2%) stage.

The common effect model demonstrated that AI models performed significantly better in prospective versus retrospective studies, in terms of sensitivity (0.98 vs 0.92; *P* < .0001), NPV (0.93 vs 0.82; *P* < .001), observed agreement (0.93 vs 0.88; *P* < .0001), and F1 score (0.95 vs 0.90; *P* < .0001). Single or bicenter studies also demonstrated better performance than multicentric in terms of specificity (0.97 vs 0.89; *P* < .0001) and NPV (0.94 vs 0.86; *P* < .0001). Furthermore, in subanalyses between adults versus pediatrics, AI models performed significantly better in adult populations for most performance metrics: sensitivity (0.97 vs 0.77; *P* < .0001), specificity (0.94 vs 0.80; *P* < .001), NPV (0.94 vs 0.52; *P* < .001), observed agreement (0.93 vs 0.74; *P* < .0001), and F1 score (0.94 vs 0.82; *P* < .0001). During the model development from training to validation, notable improvements were observed in most performance measures: sensitivity (0.92 vs 0.97; *P* < .0001), specificity (0.86 vs 0.96; *P* < .0001), NPV (0.63 vs 0.95; *P* < .0001), observed agreement (0.87 vs 0.95; *P* < .0001), and F1 score (0.91 vs 0.96; *P* < .0001).

When adjusted to the random effects model, AI models performed significantly better in prospective versus retrospective studies in terms of specificity (0.91 vs 0.82; *P* = .03). Single or bicenter studies demonstrated better performance than multicentric in terms of specificity (0.94 vs 0.84; *P* < .0001) and observed agreement (0.90 vs 0.83; *P* = .03). Furthermore, in subanalyses between adults versus pediatrics, AI models confirmed better performance in adults for specificity (0.90 vs 0.72; *P* = .03) and observed agreement (0.90 vs 0.69; *P* = .02). No significant differences were found in the subanalysis of the other aforementioned parameters.

#### 3.4.2. Meta-regression

These sub-meta-analysis findings were supported by the meta-regression results (Table 3). According to the meta-regression, pediatric AI models showed significantly lower diagnostic performance across all statistical measures compared to adult AI models, with the exception of PPV: sensitivity lower than 0.24 (−0.35, −0.12; *P* < .001), specificity lower than 0.21 (−0.33, −0.09; *P* = .0008), NPV lower than 0.49 (−0.71, −0.26; *P* < .001), observed agreement lower than 0.28 (−0.38, −0.17; *P* < .001) and F1 score lower than 0.19 (−0.30, −0.09; *P* = .0002) (Table 3).

## 4. Discussion

To the best of our knowledge, this is the first systematic review, meta-analysis, and meta-regression focused on evaluating AI models for assessing histological remission in UC and comparing digital pathology-based algorithms with pathologists. Despite the predictable substantial heterogeneity, our analysis revealed considerable AI diagnostic performance nearly comparable to that of pathologists, with nonsignificant differences in specificity, observed agreement, and F1 score. However, they outperformed AI in sensitivity and NPV.

Our results highlight the significant potential of AI in the field of digital pathology for IBD. AI has emerged as a valuable tool for precise and efficient disease assessment, crucial for clinical trials and routine clinical practice. In clinical trials, AI-enhanced digital pathology can standardize and expedite slide evaluations, reducing costs, time, and human effort.^24^ Furthermore, AI can address the challenges of intra- and interobserver variability, providing an accurate and objective assessment of the disease and contributing to consistent and reliable central read-outs in clinical trials. Similarly, AI can support pathologists in clinical practice by offering rapid and accurate disease assessment, thereby improving patient management.^25^ Notably, we developed the first convolutional neural network that accurately predicts histological remission and long-term outcomes in UC patients based solely on the presence or absence of neutrophils.^13,16^ Hence, AI can potentially guide therapeutic decision-making, paving the way for precision histology assessment.

One of the key strengths of AI models lies in their ability to assess disease activity accurately without relying on pathologists. Our meta-analysis supports this, showing that AI can achieve diagnostic performance comparable to that of pathologists. This capability is particularly valuable in clinical settings where pathologists with adequate expertise are not available, as AI can provide a more reliable and standardized alternative to nonexpert assessment. Additionally, AI tools offer significant educational benefits by highlighting algorithmically identified histological features, which can enhance diagnostic skills and improve consistency across various levels of expertise.^31^

However, AI models still require refinement,^32^ as evidenced by the significantly higher sensitivity and NPV observed in pathologists, confirmed at pairwise comparisons. This disparity likely stems from the complexity of IBD pathology, which involves diverse patterns and subtle inflammation that may be challenging for AI to detect. To address this, AI models need to be trained with diverse and high-quality prospective data that encompasses the full range of histological features necessary for accurate disease assessment. Furthermore, improving AI sensitivity can be achieved by refining algorithms and implementing more advanced AI architectures capable of capturing complex patterns in histological slides. Indeed, we have recently proposed an innovative active learning-based iterative framework that showed promise in standardizing digital tissue annotation, alleviating the burden of WSI labeling, and improving disease assessment.^33^ Besides, adopting a comprehensive, multimodal approach integrating clinical, endoscopic, and OMIC data with histology, referred to as the “endo-histo-OMIC” approach, could enhance AI performance.^11^ This multisource integrated strategy can potentially improve disease assessment and prediction of clinical outcomes and response to therapy. Finally, it is essential to emphasize that while AI is a valuable tool for supporting pathologists, it cannot fully replace human expertise. Therefore, integrating AI within a framework guided and supervised by skilled pathologists remains crucial.

This meta-analysis suffers from some limitations. The main concern is the substantial heterogeneity in AI performance metrics observed across the included studies. This variability can be attributed to several factors, elucidated by our sub-meta-analyses and meta-regressions.

First, study design plays a crucial role in influencing AI performance. Our results suggest prospective studies have lower heterogeneity than retrospective studies, as they benefit from more controlled conditions and standardized procedures, likely contributing to more consistent and reliable performance metrics.

Similarly, multicentric studies demonstrated greater heterogeneity than single or bicentric studies. This is likely due to differences in data quality and standardization practices across various locations. Nonetheless, the training, testing, and validation of AI models in a multicentric context are still useful in overcoming overfitting. Population demographics also impact AI performance, with models performing better and showing less heterogeneity in adults than in pediatric populations. This may be due to the distinct pathological features seen in children and the less comprehensive training data available for pediatric cases.^34,35^

The development stage of the AI model—training, validation, or testing—further influences both performance and heterogeneity. Our findings suggest that the training stage was associated with higher heterogeneity and lower performance. This confirms the importance of rigorous and standardized methodology potentially leading to improve the AI development process and its accuracy for patient diagnosis and outcomes. Ensuring comprehensive testing and thorough validation is crucial for enhancing the generalizability and reliability of AI models to be successfully implemented and adopted. Additionally, the comparison of different AI models influences the heterogeneity of the study. The development of AI histology models must consider the standardization of processed WSI by color, resolution, and size, to reduce the variability of AI models’ performance.

Another limitation is that only a minority of studies involved GI pathologist assessment, and some studies relied only on isolated morphological criteria rather than validated scores for predicting histological remission. Therefore, more robust and refined AI model studies should be considered to confirm these results and derive conclusive comparison data.

Finally, the absence of performance metrics for non-GI pathologists in the literature is notable, as it hindered our ability to perform a network meta-analysis. Such an analysis is essential for assessing whether AI models are at least as effective as human pathologists, thereby validating their practical applicability and usefulness, especially within the broader pathologist community.

In conclusion, our systematic review, meta-analysis, and meta-regression highlight AI’s promising potential in accurately assessing histological remission, positioning it as a valuable tool for enhancing clinical trials and IBD patient management. This prompts future research aimed at refining AI histology models to improve their performance and align more closely with pathologists. Additionally, there is a need for more standardized, large-scale studies, including randomized trials, to minimize heterogeneity and validate these preliminary findings, thereby facilitating the integration of AI into clinical practice.

## Supplementary Material

jjae198_suppl_Supplementary_Tables

## Data Availability

The data underlying this article are available in the article and in its online supplementary material. Additional data underlying this article will be shared on reasonable request to the corresponding author.
