# Supplementary material for: Artificial intelligence–enabled histology exhibits comparable accuracy to pathologists in assessing histological remission in ulcerative colitis: a systematic review, meta-analysis, and meta-regression
Source: J Crohns Colitis. 2025 Jan 1;19(1):jjae198. doi: 10.1093/ecco-jcc/jjae198 (PMC11724188; doi:10.1093/ecco-jcc/jjae198)
Supplement: jjae198_suppl_Supplementary_Tables [file jjae198_suppl_supplementary_tables.docx]

**Supplementary table 1**. General description of the included studies and contingency tables (or confusion matrices).

|  | **Studies**  **(*N*=12)** | **AI models**  **contingency tables (*N*=25)** |
| --- | --- | --- |
| **Study design** |  |  |
| Retrospective | 6 (50.0) | 12 (48.0) |
| Prospective | 6 (50.0) | 13 (52.0) |
| **Centres** |  |  |
| Single or bicentre | 5 (41.7) | 8 (32.0) |
| Multicentre | 7 (58.3) | 17 (68.0) |
| **Population** |  |  |
| Adults | 10 (83.3) | 20 (80.0) |
| Paediatrics | 2 (16.7) | 5 (20.0) |
| **Clinical score** |  |  |
| Geboes histological score (GHS) | 1 (8.3) | 2 (8.0) |
| Nancy histological index (NHI) | 4 (33.3) | 7 (28.0) |
| PICaSSO Histological remission Index (PHRI) | 4 (33.3) | 11 (44.0) |
| Other unstandardised scores | 3 (25.0) | 5 (20.0) |
| *Eosinophils* | 2/3 | 2/5 |
| *Goblet cells* | 1/3 | 3/5 |
| **Model stage** |  |  |
| Training | 3 (25.0) | 4 (16.0) |
| Testing | 6 (50.0) | 12 (48.0) |
| Validation | 3 (25.0) | 9 (36.0) |

**Supplementary table 2**. Sub-metanalysis by different study design: retrospective vs prospective studies.

| **Subgroup** | **No.  studies** | **Common effect model** | | | | | **Random effects model** | | | |
| --- | --- | --- | --- | --- | --- | --- | --- | --- | --- | --- |
|  |  | **Proportion (95% CI)** | **Q** | **I^2^ (%)** | **Q between groups (p-value)** | **Q within groups  (*p*-value)** | **Proportion (95% CI)** | **τ^2^** | **τ** | **Q between groups (p-value)** |
| **Sensitivity (recall)** | | | | | | | | | | |
| **Retrospective** | 12 | 0.9175  (0.9051-0.9299) | 338.14 | 96.7 | 56.61 | 460.56 | 0.8123  (0.7254-0.8992) | 0.021 | 0.1449 | 1.6 |
| **Prospective** | 13 | 0.9754  (0.9668-0.984) | 122.42 | 90.2 | (<.0001) | (<.0001) | 0.874  (0.8341-0.914) | 0.0035 | 0.0588 | (.2055) |
| **Specificity** | | | | | | | | | | |
| **Retrospective** | 12 | 0.9423  (0.9274-0.9572) | 153.98 | 92.9 | 1.27 | 205.02 | 0.8208  (0.7475-0.8942) | 0.0137 | 0.117 | 4.51 |
| **Prospective** | 13 | 0.9304  (0.916-0.9448) | 51.04 | 76.5 | (.26) | (<.0001) | 0.9081  (0.8749-0.9413) | 0.0024 | 0.0492 | (.0337) |
| **PPV (precision)** | | | | | | | | | | |
| **Retrospective** | 12 | 0.9594  (0.9495-0.9693) | 113.48 | 90.3 | 3.97 | 173.43 | 0.8907  (0.8459-0.9355) | 0.0046 | 0.0677 | 0.03 |
| **Prospective** | 13 | 0.943  (0.9303-0.9557) | 59.95 | 80 | (.0464) | (<.0001) | 0.8962  (0.8589-0.9334) | 0.003 | 0.0552 | (.8552) |
| **NPV** | | | | | | | | | | |
| **Retrospective** | 12 | 0.8236  (0.8074-0.8399) | 1173.34 | 99.1 | 92.91 | 1230.61 | 0.7148  (0.5313-0.8982) | 0.1023 | 0.3198 | 3.52 |
| **Prospective** | 13 | 0.9308  (0.9163-0.9453) | 57.27 | 79 | (<.0001) | (<.0001) | 0.8938  (0.8572-0.9303) | 0.003 | 0.0544 | (.0607) |
| **Observed agreement (accuracy)** | | | | | | | | | | |
| **Retrospective** | 12 | 0.8843  (0.8723-0.8964) | 354.22 | 96.9 | 31.97 | 442.04 | 0.8211  (0.7466-0.8956) | 0.0164 | 0.1281 | 2.42 |
| **Prospective** | 13 | 0.9297  (0.9196-0.9398) | 87.82 | 86.3 | (<.0001) | (<.0001) | 0.8857  (0.8529-0.9186) | 0.0028 | 0.0526 | (.1197) |
| **F1 score** | | | | | | | | | | |
| **Retrospective** | 12 | 0.9044  (0.8943-0.9145) | 272.84 | 96 | 46.9 | 419.79 | 0.8375  (0.7777-0.8973) | 0.0099 | 0.0996 | 0.7 |
| **Prospective** | 13 | 0.9503  (0.9419-0.9587) | 146.95 | 91.8 | (<.0001) | (<.0001) | 0.868  (0.8286-0.9074) | 0.0041 | 0.0639 | (.4039) |

**Supplementary table 3**. Sub-metanalysis by different number of participating centres: single or bicentric vs multicentric studies.

| **Subgroup** | **No.  studies** | **Common effect model** | | | | | **Random effects model** | | | |
| --- | --- | --- | --- | --- | --- | --- | --- | --- | --- | --- |
|  |  | **Proportion (95% CI)** | **Q** | **I^2^ (%)** | **Q between groups (p-value)** | **Q within groups  (*p*-value)** | **Proportion (95% CI)** | **τ^2^** | **τ** | **Q between groups (p-value)** |
| **Sensitivity (recall)** | | | | | | | | | | |
| **Single or  bicentre** | 8 | 0.8953  (0.8662-0.9244) | 20.91 | 66.5 | 18.06 | 499.11 | 0.8644  (0.8074-0.9215) | 0.0039 | 0.0625 | 0.7 |
| **Multicentre** | 17 | 0.9604  (0.9531-0.9677) | 478.2 | 96.7 | (<.0001) | (<.0001) | 0.8326  (0.7844-0.8807) | 0.0084 | 0.0918 | (.4029) |
| **Specificity** | | | | | | | | | | |
| **Single or  bicentre** | 8 | 0.9738  (0.9601-0.9876) | 32.93 | 78.7 | 67.04 | 139.24 | 0.9415  (0.9038-0.9793) | 0.0019 | 0.0434 | 11.21 |
| **Multicentre** | 17 | 0.8865  (0.8708-0.9023) | 106.31 | 85 | (<.0001) | (<.0001) | 0.8409  (0.7957-0.8861) | 0.0066 | 0.0813 | (<.0001) |
| **PPV (precision)** | | | | | | | | | | |
| **Single or  bicentre** | 8 | 0.9332  (0.9087-0.9577) | 15.33 | 54.3 | 2.86 | 174.53 | 0.9231  (0.8836-0.9627) | 0.0016 | 0.0399 | 2.25 |
| **Multicentre** | 17 | 0.9555  (0.9472-0.9637) | 159.2 | 89.9 | (.0905) | (<.0001) | 0.8835  (0.85-0.917) | 0.0034 | 0.0586 | (.1336) |
| **NPV** | | | | | | | | | | |
| **Single or  bicentre** | 8 | 0.9427  (0.9233-0.9621) | 44.78 | 84.4 | 51.92 | 1271.6 | 0.8844  (0.8247-0.944) | 0.0055 | 0.0742 | 2.91 |
| Multicentre | 17 | 0.8568  (0.8437-0.8698) | 1226.82 | 98.7 | (<.0001) | (<.0001) | 0.767  (0.6463-0.8878) | 0.0616 | 0.2482 | (.0878) |
| **Observed agreement (accuracy)** | | | | | | | | | | |
| **Single or  bicentre** | 8 | 0.9226  (0.9054-0.9399) | 30.39 | 77 | 2.16 | 471.85 | 0.9009  (0.8623-0.9395) | 0.0022 | 0.0474 | 4.7 |
| **Multicentre** | 17 | 0.9081  (0.8995-0.9168) | 441.46 | 96.4 | (.1415) | (<.0001) | 0.8306  (0.7801-0.8811) | 0.0103 | 0.1014 | (.0302) |
| **F1 score** | | | | | | | | | | |
| **Single or  bicentre** | 8 | 0.8942  (0.8729-0.9155) | 13.65 | 48.7 | 13.13 | 453.56 | 0.8846  (0.8522-0.9169) | 0.001 | 0.031 | 2.58 |
| **Multicentre** | 17 | 0.9355  (0.9287-0.9422) | 439.92 | 96.4 | (<.001) | (<.0001) | 0.8415  (0.8002-0.8829) | 0.0065 | 0.0809 | (.1082) |

**Supplementary table 4**. Sub-metanalysis by different population age group: adults vs paediatrics.

| **Subgroup** | **No.  studies** | **Common effect model** | | | | | **Random effects model** | | | |
| --- | --- | --- | --- | --- | --- | --- | --- | --- | --- | --- |
|  |  | **Proportion (95% CI)** | **Q** | **I^2^ (%)** | **Q between groups (p-value)** | **Q within groups  (*p*-value)** | **Proportion (95% CI)** | **τ^2^** | **τ** | **Q between  groups (p-value)** |
| **Sensitivity (recall)** | | | | | | | | | | |
| **Adults** | 20 | 0.9697  (0.9623-0.977) | 142.85 | 86.7 | 184.87 | 332.3 | 0.8957  (0.8678-0.9236) | 0.0023 | 0.0478 | 3.77 |
| **Paediatrics** | 5 | 0.7734  (0.7461-0.8007) | 189.45 | 97.9 | (<.0001) | (<.0001) | 0.6961  (0.4966-0.8956) | 0.0501 | 0.2238 | (.0522) |
| **Specificity** | | | | | | | | | | |
| **Adults** | 20 | 0.9436  (0.9329-0.9542) | 120.5 | 84.2 | 35.51 | 170.77 | 0.9043  (0.8732-0.9355) | 0.0035 | 0.059 | 4.52 |
| **Paediatrics** | 5 | 0.8024  (0.7572-0.8476) | 50.27 | 92 | (<.0001) | (<.0001) | 0.7202  (0.5534-0.8871) | 0.0326 | 0.1806 | (.0335) |
| **PPV (precision)** | | | | | | | | | | |
| **Adults** | 20 | 0.9362  (0.9258-0.9465) | 66.94 | 71.6 | 24.38 | 153.02 | 0.9112  (0.8869-0.9355) | 0.0017 | 0.0406 | 2.01 |
| **Paediatrics** | 5 | 0.9759  (0.964-0.9879) | 86.08 | 95.4 | (<.0001) | (<.0001) | 0.8219  (0.701-0.9428) | 0.0172 | 0.1313 | (.156) |
| **NPV** | | | | | | | | | | |
| **Adults** | 20 | 0.9354  (0.9238-0.9469) | 93.45 | 79.7 | 611.03 | 712.49 | 0.8955  (0.8656-0.9254) | 0.0029 | 0.0542 | 3.11 |
| **Paediatrics** | 5 | 0.5243  (0.4938-0.5548) | 619.04 | 99.4 | (<.0001) | (<.0001) | 0.524  (0.1121-0.9358) | 0.2185 | 0.4675 | (.0778) |
| **Observed agreement (accuracy)** | | | | | | | | | | |
| **Adults** | 20 | 0.9284  (0.9202-0.9365) | 108.73 | 82.5 | 188.86 | 285.15 | 0.8985  (0.8761-0.921) | 0.0018 | 0.0427 | 5.34 |
| **Paediatrics** | 5 | 0.7409  (0.7154-0.7664) | 176.42 | 97.7 | (<.0001) | (<.0001) | 0.6899  (0.5143-0.8654) | 0.039 | 0.1975 | (.0208) |
| **F1 score** | | | | | | | | | | |
| **Adults** | 20 | 0.9446  (0.9378-0.9514) | 159.52 | 88.1 | 128.91 | 337.79 | 0.8891  (0.864-0.9141) | 0.0022 | 0.0471 | 3.79 |
| **Paediatrics** | 5 | 0.8239  (0.8042-0.8436) | 178.26 | 97.8 | (<.0001) | (<.0001) | 0.7365  (0.5851-0.8879) | 0.0289 | 0.1699 | (.0514) |

**Supplementary table 5**. Sub-metanalysis by different model stage: training vs testing vs validation.

| **Subgroup** | **No.  studies** | **Common effect model** | | | | | **Random effects model** | | | |
| --- | --- | --- | --- | --- | --- | --- | --- | --- | --- | --- |
|  |  | **Proportion (95% CI)** | **Q** | **I^2^ (%)** | **Q between groups (p-value)** | **Q within groups  (*p*-value)** | **Proportion (95% CI)** | **τ^2^** | **τ** | **Q between  groups (p-value)** |
| **Sensitivity (recall)** | | | | | | | | | | |
| **Training** | 4 | 0.9186  (0.9039-0.9333) | 252.97 | 98.8 | 33.71 | 483.46 | 0.7563  (0.5372-0.9753) | 0.0471 | 0.217 | 3.89 |
| **Testing** | 12 | 0.9699  (0.9588-0.981) | 188.94 | 94.2 | (<.0001) | (<.0001) | 0.8268  (0.7566-0.8969) | 0.013 | 0.114 | (.1428) |
| **Validation** | 9 | 0.9659  (0.9542-0.9777) | 41.54 | 80.7 |  |  | 0.8989  (0.8512-0.9466) | 0.0034 | 0.0579 |  |
| **Specificity** | | | | | | | | | | |
| **Training** | 4 | 0.8558  (0.821-0.8907) | 30.72 | 90.2 | 31.36 | 174.93 | 0.8259  (0.6977-0.954) | 0.0146 | 0.1208 | 0.63 |
| **Testing** | 12 | 0.9259  (0.9099-0.942) | 81.35 | 86.5 | (<.0001) | (<.0001) | 0.881  (0.833-0.9291) | 0.0055 | 0.0743 | (.7293) |
| **Validation** | 9 | 0.9591  (0.9444-0.9739) | 62.86 | 87.3 |  |  | 0.8773  (0.8203-0.9342) | 0.0052 | 0.0719 |  |
| **PPV (precision)** | | | | | | | | | | |
| **Training** | 4 | 0.9692  (0.9585-0.98) | 44.45 | 93.3 | 34.46 | 142.93 | 0.9315  (0.8701-0.9929) | 0.003 | 0.0552 | 2.16 |
| **Testing** | 12 | 0.9017  (0.8819-0.9216) | 64.4 | 82.9 | (<.0001) | (<.0001) | 0.8726  (0.8217-0.9234) | 0.0063 | 0.0792 | (.3399) |
| **Validation** | 9 | 0.9518  (0.9379-0.9657) | 34.08 | 76.5 |  |  | 0.9037  (0.8613-0.9462) | 0.0024 | 0.0495 |  |
| **NPV** | | | | | | | | | | |
| **Training** | 4 | 0.6262  (0.5991-0.6533) | 726.79 | 99.6 | 422.65 | 900.87 | 0.5734  (0.083-1.0638) | 0.2483 | 0.4983 | 1.85 |
| **Testing** | 12 | 0.9107  (0.8935-0.928) | 121.79 | 91 | (<.0001) | (<.0001) | 0.854  (0.7921-0.9158) | 0.0101 | 0.1007 | (.3959) |
| **Validation** | 9 | 0.9513  (0.9351-0.9675) | 52.28 | 84.7 |  |  | 0.8821  (0.8289-0.9352) | 0.0043 | 0.0654 |  |
| **Observed agreement (accuracy)** | | | | | | | | | | |
| **Training** | 4 | 0.8654  (0.849-0.8819) | 204.86 | 98.5 | 75.77 | 398.23 | 0.7788  (0.5992-0.9584) | 0.0327 | 0.1807 | 2.26 |
| **Testing** | 12 | 0.8893  (0.8752-0.9034) | 131.77 | 91.7 | (<.0001) | (<.0001) | 0.8561  (0.8053-0.907) | 0.0072 | 0.0846 | (.3229) |
| **Validation** | 9 | 0.9459  (0.9347-0.9571) | 61.6 | 87 |  |  | 0.8914  (0.8509-0.9318) | 0.0029 | 0.054 |  |
| **F1 score** | | | | | | | | | | |
| **Training** | 4 | 0.9094  (0.8974-0.9214) | 162.91 | 98.2 | 51.25 | 415.44 | 0.823  (0.7036-0.9423) | 0.0136 | 0.1167 | 2.09 |
| **Testing** | 12 | 0.9093  (0.896-0.9225) | 184.93 | 94.1 | (<.0001) | (<.0001) | 0.8395  (0.7787-0.9002) | 0.0103 | 0.1016 | (.3513) |
| **Validation** | 9 | 0.9565  (0.9471-0.9658) | 67.59 | 88.2 |  |  | 0.8866  (0.8431-0.93) | 0.0032 | 0.0568 |  |
